# Eucalyptus in Actinomycosis

**Published:** 1898-06-04

**Authors:** 


### EUCALYPTUS IN ACTINOMYCOSIS.

A good case is reported by Dr. Glentworth Butler in the New York *Medical News*, in which recovery from pulmonary actinomycosis took place under the use of eucalyptus oil. The patient was a male, aged 37 years, who for some weeks had had a cough and dark-coloured expectoration. He was brought to hospital for an injury, having been struck on the head by a falling plank and thrown into the water. On admission, he was found to be suffering from a scalp wound and a compound fracture of the nasal bones. A week afterwards he began to have pain in the right side and to cough, his temperature rose, and his respiration became rapid. Some dulness was found over the base of the right lung, with subcrepital râles, which later on became bubbling in character and were also found at the left apex, which became dull down to the nipple. For six or seven weeks the temperature rose and fell remittently, and there were profuse sweatings like those of pyæmia. Violent and prolonged paroxysms of cough came on, with the discharge of extremely offensive dark brown sputum. These attacks of coughing were often precipitated by change of position. The breath became almost continuously offensive. Oil of eucalyptus was given, both in capsules (five minims every four hours) and as a spray inhalation three times a day. Examination of the sputum at first gave only negative results, but about five weeks after the accident examination resulted in the discovery of the organism of actinomycosis. In the meanwhile, under the use of the eucalyptus, the expectoration had been becoming less offensive, so that it had only the odour of eucalyptus. Nine weeks after admission sleep, appetite, and digestion were all that could be desired, the cough and expectoration had entirely disappeared, and the pulse, the temperature, and the respiration were normal. There was still, however, slight dulness over the left apex, with high-pitched breathing and prolonged expiration in the infra-clavicular space, and a similar dulness with a feeble respiratory murmur and prolonged expiration at the right base, but no râles in either locality. This result seems to be good so far as it goes, especially in view of the high mortality which is met with among those cases of pulmonary disease in which the organism has actually been found. The vitality of the ray-fungus does not appear to be very high, if only one could but get at it, and there seems to be but little doubt that the oil of eucalyptus is excreted by the pulmonary mucous membrane—which is coming pretty near, although perhaps not quite to the point where it is wanted.

